# JMJD1B, a novel player in histone H3 and H4 processing to ensure genome stability

**DOI:** 10.1186/s13072-020-00331-1

**Published:** 2020-02-18

**Authors:** Francisco Saavedra, Zachary A. Gurard-Levin, Camila Rojas-Villalobos, Isabelle Vassias, Raquel Quatrini, Geneviève Almouzni, Alejandra Loyola

**Affiliations:** 1 Fundación Ciencia & Vida, 7780272 Santiago, Chile; 2 Universidad San Sebastián, 7510156 Santiago, Chile; 3 CNRS, UMR3664, Equipe Labellisée Ligue contre le Cancer, Institut Curie, PSL Research University, Paris, 75005 France; 4 UPMC Univ Paris 06, CNRS, UMR3664, Sorbonne Universités, Paris, 75005 France; 5 Present Address: SAMDI Tech, Inc, Chicago, IL 60616 USA

**Keywords:** Newly synthesized histones, Genomic instability, Histone H3, Histone processing, JMJD1B, NASP, Histone supply, Cancer, Histone maturation

## Abstract

**Background:**

Maintaining a proper supply of soluble histones throughout the cell cycle is important to ensure chromatin and genome stability. Following their synthesis, histones undergo a series of maturation steps to prepare them for deposition onto chromatin.

**Results:**

Here, we identify the lysine demethylase JMJD1B as a novel player in the maturation cascade that contributes to regulate histone provision. We find that depletion of JMJD1B increases the protein levels of the histone chaperone tNASP leading to an accumulation of newly synthesized histones H3 and H4 at early steps of the histone maturation cascade, which perturbs chromatin assembly. Furthermore, we find a high rate of JMJD1B mutations in cancer patients, and a correlation with genomic instability.

**Conclusions:**

Our data support a role for JMJD1B in fine-tuning histone supply to maintain genome integrity, opening novel avenues for cancer therapeutics.

## Background

Histone synthesis is tightly coupled to DNA replication and peaks during the G1/S phase transition to account for doubling the genome. At this time, canonical histone genes are expressed, in contrast to histone variant genes, that are expressed at basal levels throughout the cell cycle [21, 22]. Outside S-phase a histone pool is maintained in case of unanticipated changes to histone demand, such as DNA damage [9]. Maintaining a proper level of soluble histones is critical. While depleting the histone pool induces profound cell growth defects such as sensitivity to DNA damage, S-phase slow down and G2/M arrest [12, 24], excess histones also have deleterious effects as shown in yeast [32]. Cells therefore utilize various mechanisms to ensure proper histone provision, including degrading excess histones by the proteasome and chaperone mediated-autophagy in yeast and mammalian cells, respectively [5, 12].

Following biosynthesis, histones H3 and H4 proceed through a maturation cascade to acquire their correct folding and to establish post-translational modifications [1, 3, 7–9, 30, 31]. In this processing pathway histones H3 and H4 are escorted by chaperones such as Hsp90, Hsc70, Hsp70; histone chaperones such as NASP, Asf1, RbAp48; and histone interacting proteins, such as PP32/SET, HAT1, and Importin4 [1, 3, 30]. Newly synthesized histone H3 is monomethylated at lysine 9 (H3K9me1) during translation by the enzyme SetDB1 that is able to associate with ribosomes [28]. At chromatin, H3K9me1 can be further methylated by the Suv39H1 methyltransferase to H3K9me3, which recruits heterochromatin protein 1 (HP1) during heterochromatin replication [20, 23, 27, 33] to ensure genome stability. Newly synthesized histone H4, on the other hand, is acetylated at both lysine 5 and 12 by HAT1 in a timely regulated manner. Thus, PP32/SET proteins protect acetylation of newly synthesized histone H4 to regulate the timing of acetylation [30].

Here, we identify the histone demethylase JMJD1B as a novel player in newly synthesized histone metabolism. Specifically, we find that if JMJD1B is not present, chromatin assembly defects and DNA damage are observed. At the molecular level, we find that upon reducing the JMJD1B levels, histones H3 and H4 accumulate at early stages of the processing pathway due to an accumulation of the histone chaperone tNASP. Notably, JMJD1B is mutated in several cancers. Therefore, we propose a working model where JMJD1B ensures histone supply and thereby impacts genome integrity.

## Results

### Cells treated with siJMJD1B accumulate soluble histones H3 and the histone chaperone tNASP

**Fig. 1:**
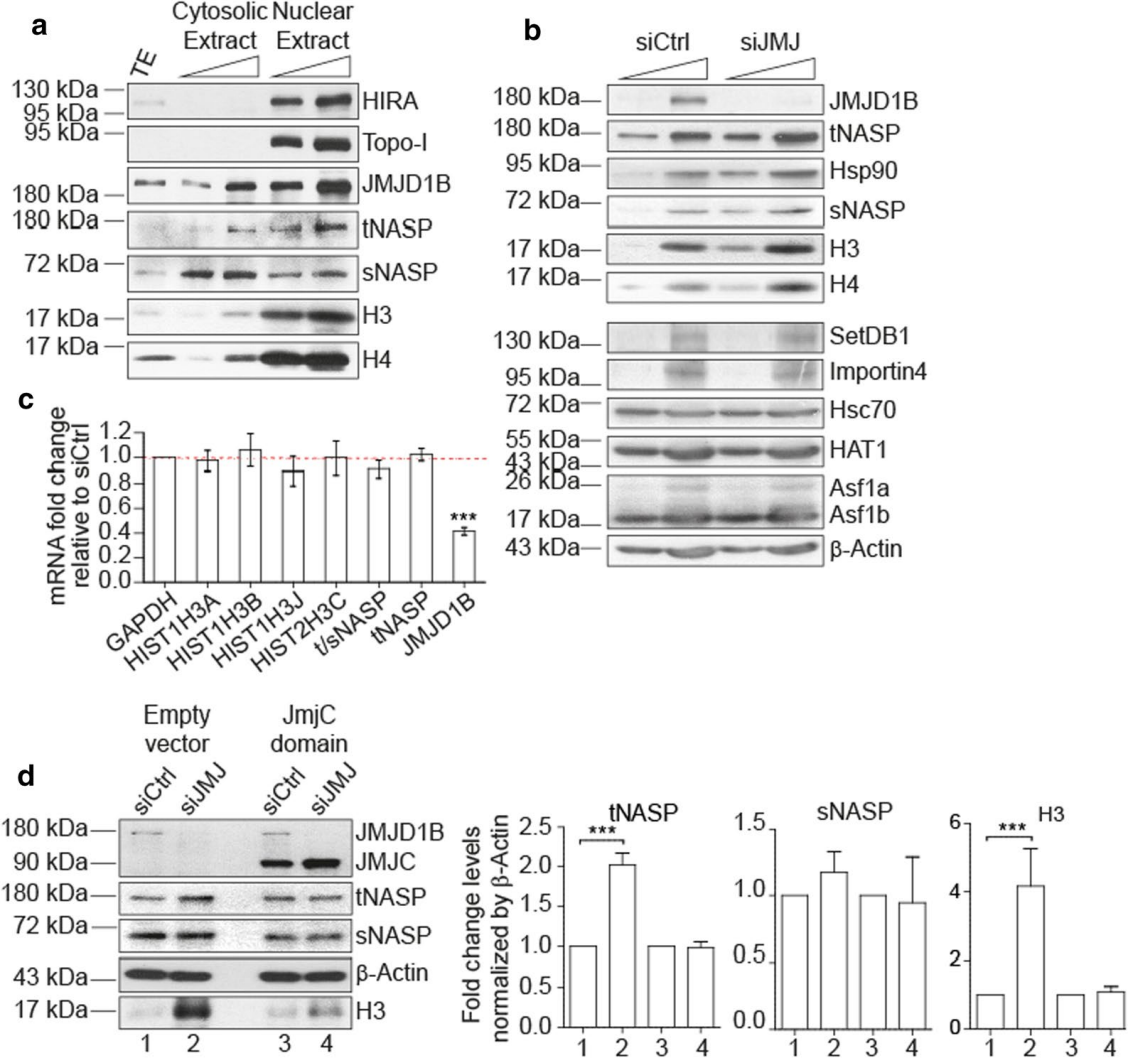
siJMJD1B cells accumulate tNASP, Hsp90, H3, and H4. **a** Western blot of 10 and 20 µg of cytosolic and nuclear extracts derived from HeLa cells. As control of the extract preparation, HIRA and Topo-I were analyzed as nuclear extract markers, showing that cytosolic extract is not contaminated with nuclear components. TE, total cell extract derived from HeLa cells. **b** Western blot of 5 and 15 µg of cytosolic extracts derived from either siControl or siJMJD1B treated HeLa cells. **c** Levels of mRNA from samples derived from siControl or siJMJD1B treated HeLa cells measured by real-time PCR. The graph shows the fold change of the different mRNAs relative to siControl. Each expression level was normalized to that of GAPDH. The standard deviation was obtained from three independent experiments. ***p < 0.001, Student’s *t*-test. **d** Cells were treated with either siControl or siJMJD1B for 48 h. Then, cells were treated with either empty or the JMJD1B derived JmjC-domain containing vector, for 24 h. Cytosolic extracts were prepared and analyzed by Western blot. To the left, Western blot of 20 µg of cytosolic extracts derived from either siControl or siJMJD1B treated HeLa cells that have been transcomplemented with either empty vector or JmjC-domain. To the right, graphs showing the quantitation of tNASP, sNASP, and histone H3 normalized by β-actin. The standard deviation was obtained from three independent experiments. ***p < 0.001, Student’s *t*-test

Our lab previously described that SetDB1 establishes H3K9me1 during translation [28]. We further characterized SetDB1 interactions by mass spectrometry from cytosolic extracts derived from HeLa cells and, in addition to known players in metabolizing newly synthesized histones, we identified a new interacting partner, the demethylase JMJD1B (also referred to as KDM3B), a candidate tumor suppressor [10] with H3K9me1/2 and H4R3me2 demethylase activities [2, 16] (Additional file 1: Figure S1). We investigated its cellular localization and found it in both cytosolic and nuclear extracts derived from HeLa cells (Fig. 1a). We therefore asked what role JMJD1B plays in newly synthesized histone maturation by exploiting the knockdown of JMJD1B by treating HeLa cells with siRNA against JMJD1B (siJMJD1B) and observed the reduction of both JMJD1B protein on cytosolic extracts (Fig. 1b) and mRNA levels (Fig. 1c) 72 h after the treatment. Remarkably, upon siJMJD1B we observed an increase in the cytosolic histones H3 and H4 protein levels (Fig. 1b). This accumulation was not due to changes in gene expression, since H3 mRNA levels did not change upon siJMJD1B (Fig. 1c). Histone chaperones of the early histone H3 maturation complexes, including tNASP and Hsp90, also accumulated (Fig. 1b). Accumulation of NASP was not due to changes in gene expression, as its mRNA levels do not change (Fig. 1c). In contrast, other proteins that interact with newly synthesized histone H3 did not change their protein levels, including SetDB1, Importin4, Hsc70, HAT1, Asf1a/b (Fig. 1b), and sNASP (Fig. 1d, second graph, compare lanes 1 and 2). To investigate whether the demethylase activity of JMJD1B participates in the H3 maturation process, we performed a transcomplementation assay on siJMJD1B treated cells and found that upon decreasing the levels of JMJD1B, cytosolic histone H3 and tNASP accumulate (Fig. 1d, lanes 1 and 2). However, cytosolic H3 and tNASP are not accumulated in cells transcomplemented with the catalytic JmjC-domain of JMJD1B 48 h after the siJMJD1B treatment (Fig. 1d, lanes 3 and 4). Unexpectedly, upon siJMJD1B cytosolic H3K9me1 levels are decreased (data not shown), suggesting that JMJD1B is acting on non-histone substrate. Therefore, these data show that upon siJMJD1B treatment, components of the early H3 maturation cascade complexes accumulate in cytosolic extracts, including tNASP, Hsp90, H3, and H4.

### tNASP overexpression mimics the H3 accumulation observed upon siJMJD1B

**Fig. 2:**
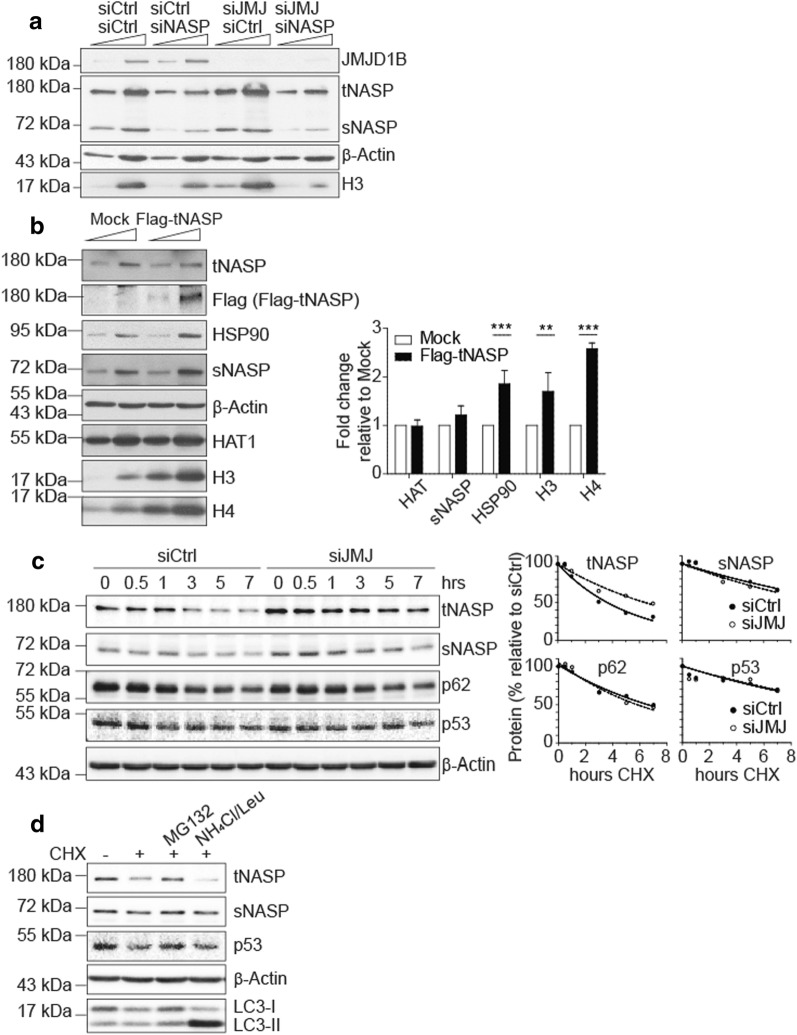
tNASP overexpression mimics the H3 accumulation observed upon siJMJD1B. **a** Western blot of 5 and 15 µg of cytosolic extracts derived from siControl, siNASP, and/or siJMJD1B treated HeLa cells. **b** Western blot of 5 and 10 µg of cytosolic extracts derived from Mock or overexpressed-Flag-tNASP HeLa cells. The graph shows the fold change of proteins in the Flag-tNASP conditions, respect to Mock, normalized by β-actin. The standard deviation was obtained from three independent experiments. **p < 0.01, ***p < 0.001, Student’s *t*-test. **c** Western blot of 20 µg of total cell extracts derived from siControl or siJMJD1B HeLa cells that were treated for 0 to 7 h with 100 µg/mL of cycloheximide (CHX). The graphs show the mean value of each protein, normalized by β-actin at each time-point, from three independent experiments. **d** Western blot of 20 µg of total cell extracts derived from HeLa cells treated with vehicle (DMSO), 100 µg/mL CHX, or 100 µg/mL CHX plus protein-degradation inhibitors: 50 µM MG132 (ubiquitin–proteasome pathway) or 20 mM NH_4_Cl + 200 µM leupeptin (lysosomal pathways)

Given that siJMJD1B leads to increasing tNASP protein levels and previous data showed that NASP protects cytosolic histone H3 from degradation [5], we examined whether the accumulation of H3 observed upon siJMJD1B is dependent on the accumulation of tNASP. For this, we performed a double knock-down of JMJD1B and NASP (Fig. 2a). We observe that upon siNASP, both tNASP and sNASP protein levels diminish, JMJD1B levels do not change, and H3 levels diminish, as previously described [3, 5]. While siJMJD1B treatment accumulates both H3 and tNASP, H3 does not accumulate upon siJMJD1B in the absence of tNASP, arguing that tNASP is required for H3 accumulation. We next investigated whether overexpression of tNASP could mimic the siJMJD1B phenotype. We performed Western blot analyses and observe that when tNASP is overexpressed, Hsp90, H3, and H4 accumulate (Fig. 2b). In contrast, HAT1, sNASP, or β-actin protein levels do not change.

We then explored why tNASP accumulates upon siJMJD1B. Since tNASP mRNA levels are not changed (Fig. 1d), we analyzed tNASP degradation. By incubating HeLa cells with cycloheximide for 0–7 h, we determined that the half-life of tNASP is 3.6 h (Fig. 2c). In contrast, the tNASP half-life increases to 6.4 h upon siJMJD1B (Fig. 2c), suggesting that JMJD1B reduction leads to a defect in tNASP degradation. This degradation defect is tNASP specific, as the sNASP half-life does not change (Fig. 2c). Importantly, both ubiquitin–proteasomal and lysosomal degradation pathways are not affected upon siJMJD1B, as p53 (proteasomal degradation target) and p62 (lysosomal degradation target) half-lives are not affected upon siJMJD1B (Fig. 2c). We then characterized how tNASP is degraded by incubating HeLa cells with cycloheximide and proteasome (MG132) or lysosome (NH_4_Cl/leupeptin) inhibitors. Upon proteasomal inhibition, p53 and tNASP are not degraded, whereas NH_4_Cl/leupeptin increases the LC3-II/LC3-I ratio, and tNASP is degraded (Fig. 2d). Therefore, we conclude that the ubiquitin–proteasome pathway is the primary mechanism for tNASP degradation (Fig. 2d). Our data strongly suggest that siJMJD1B blocks the tNASP ubiquitin–proteasome degradation pathway by an as yet uncharacterized mechanism, leading to the accumulation of cytosolic histone H3.

### siJMJD1B cells accumulate histones H3 and H4 at early steps of the maturation cascade associated with tNASP/Hsp90

**Fig. 3:**
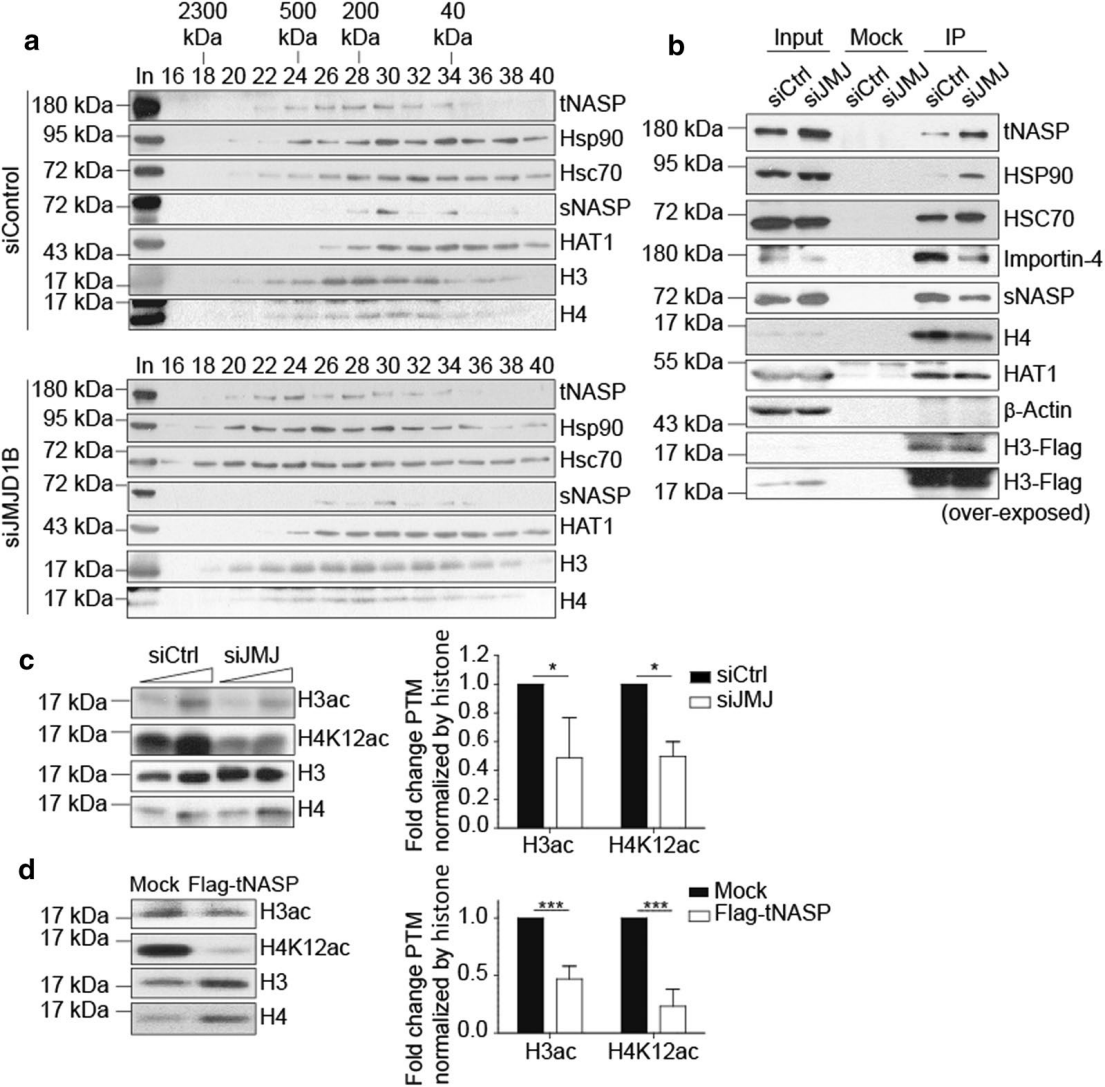
siJMJD1B cells accumulate histones H3 and H4 at early steps of the maturation cascade associated with tNASP/Hsp90/Hsc70. **a** Western blot of fractions derived from the sizing exclusion chromatography S200, loaded with 0.2 mg of cytosolic extracts derived from siControl or siJMJD1B HeLa cells. The corresponding molecular size of each fraction is specified at the top of the western blot. **b** Western blot of samples derived from immunoprecipitation assays using 1.5 mg of cytosolic extracts derived from siControl or siJMJD1B HeLa cells that express exogenously low levels of Flag-H3.1 protein. Immunoprecipitation was performed using antibodies against Flag. Input corresponds to 15 µg of cytosolic extracts. **c** Left, Western blot of 20 and 40 µg of cytosolic extracts derived from siControl and of 13 and 26 µg of cytosolic extracts from siJMJD1B HeLa cells. These amounts of cytosolic proteins correspond to equal amounts of histone H3. Right, the graph shows the quantitation of global H3ac and H4K12ac, normalized by either the amount of histone H3 or H4. The data is showed as fold change with respect to siControl. The standard deviation was obtained from three independent experiments. *p < 0.05, Student’s *t*-test. **d** Left, Western blot of 20 µg of cytosolic extracts derived from Mock or overexpressed-Flag-tNASP HeLa cells. Right, the graph shows the quantitation of global H3ac and H4K12ac, normalized by either the amount of histone H3 or H4. The data is showed as fold change with respect to Mock. The standard deviation was obtained from three independent experiments. ***p < 0.001, Student’s *t*-test

To biochemically characterize histone H3 complexes accumulated upon siJMJD1B treatment, we fractionated cytosolic extracts derived from untreated and siJMJD1B HeLa cells by size-exclusion chromatography. In control cytosolic extracts, histone H3 elutes with a molecular weight of about 250 kDa (fractions 26–28, Fig. 3a, top), as previously described [1, 3]. tNASP, Hsp90, Hsc70, and histone H4 elute in these fractions. Another complex composed of sNASP, HAT1, H3 and H4 elutes with a molecular weight of about 100 kDa (fractions 30–32). Remarkably, in siJMJD1B derived cytosolic extracts, the complex composed of tNASP, Hsp90, Hsc70, histones H3 and H4 is shifted to a heavier molecular size fraction, comprising fractions 22–24, of about 600 kDa (Fig. 3a, bottom). Although we cannot formally exclude the formation of a new cytosolic H3 complex upon siJMJD1B, we favor a model in which the higher molecular weight complex is the result of the buffering of accumulated histones H3 and H4 by tNASP, Hsp90, and Hsc70. To test this hypothesis, we performed histone H3 immunoprecipitation experiments with cytosolic extracts derived from siControl and siJMJD1B cells. To improve the immunoprecipitation efficiency, we used HeLa cells expressing low levels of Flag-H3 (Fig. 3b). Flag-H3 immunoprecipitation from siControl cells reveal the interaction of H3 with components of its maturation cascade: tNASP, Hsp90, Hsc70, Importin4, sNASP, H4, and HAT1 (Fig. 3b). As a control, β-actin, a protein that does not interact with histone H3, was not immunoprecipitated. Interestingly, in the Flag-H3 immunoprecipitation from siJMJD1B cytosolic extracts, we observed a clear shift in the H3 interaction towards tNASP, Hsp90, and Hsc70, compared to Importin4, sNASP, and HAT1 (Fig. 3b). Thus, the results strongly suggest that upon siJMJD1B histone H3 accumulates with tNASP, Hsp90, and Hsc70 chaperones in a larger molecular weight complex, at early steps of its maturation cascade.

To investigate this further, we analyzed post-translational modifications of soluble histones H3 and H4 (Fig. 3c). Newly synthesized histones H3 and H4 display small quantity of modifications that are imposed along their maturation cascade [11, 19]. The mark H3K9me1 is imposed at the ribosome [28], H3ac is imposed at Complex II [1, 3], and acetylation of histone H4 lysine 5 and 12 occurs in Complex III [1, 3]. Interestingly, upon siJMJD1B, the levels of global H3ac and H4K12ac show a marked decrease when compared to control (Fig. 3c). This is consistent with an arrest of histones H3 and H4 at early steps in the maturation pathway. When overexpressing tNASP, we observe the same effects on histone post-translational modifications: reduction of H3ac and H4ac (Fig. 3d). Taken together, we conclude that accumulation of tNASP, either by siJMJD1B or by overexpressing tNASP, leads to an increase of the soluble pool of cytosolic histones H3 and H4 at early steps of the histone maturation pathway.

### Histone deposition is affected upon siJMJD1B

**Fig. 4:**
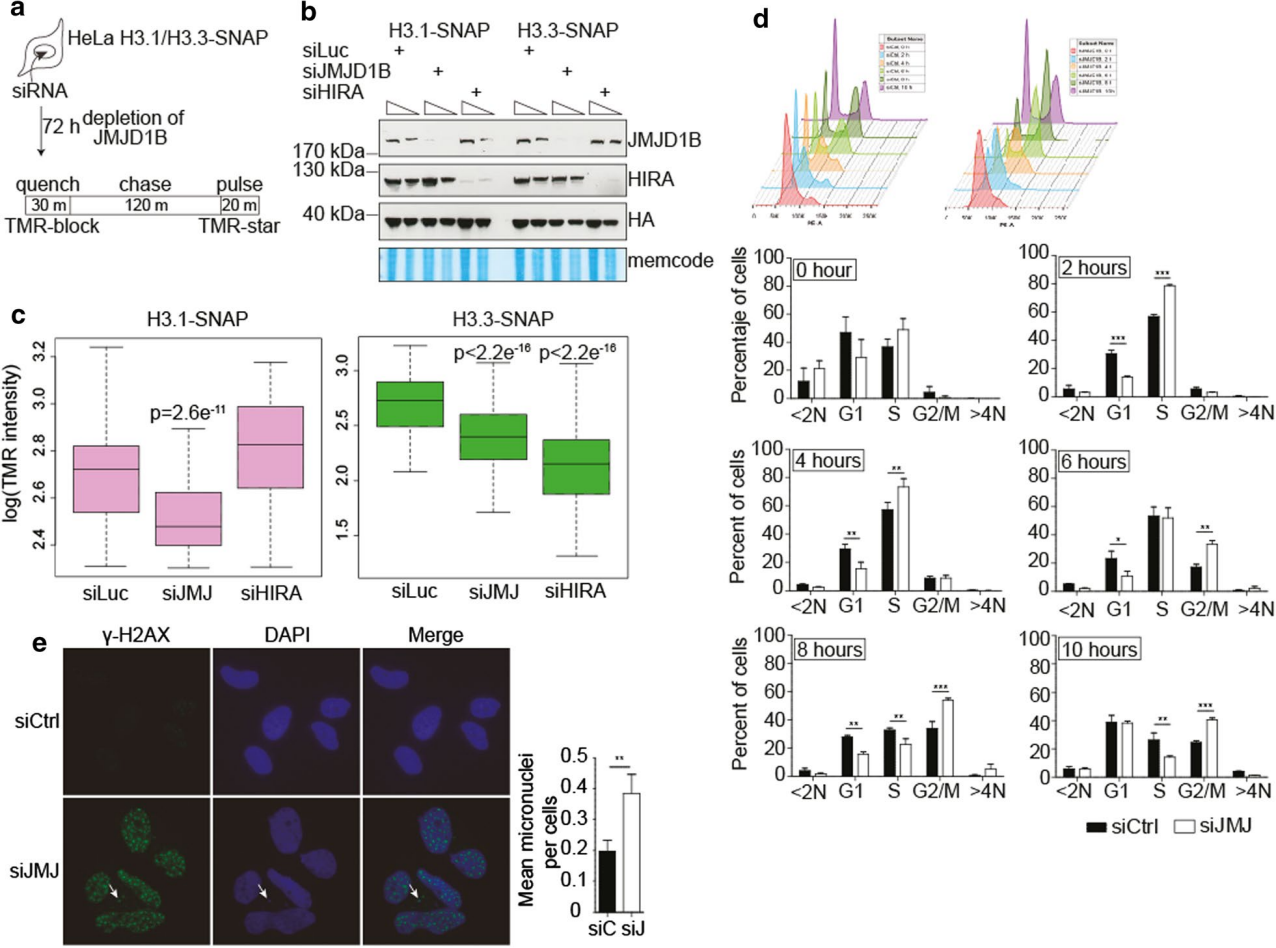
Histone deposition defects, G2/M cell cycle arrest, and DNA damage are induced upon siJMJD1B. **a** Scheme illustrating the H3.1- and H3.3-SNAP for in vivo labeling assays with red fluorescent TMR-Star in quench-chase-pulse experiments to label newly H3-SNAP synthesized during the 2 h chase. **b** Western blot of crude cell extracts from siLuciferase (siLuc), siJMJD1B, and siHIRA treated cells. **c** Box-and-whisker plot of the TMR intensity in the nucleus of HeLa cells for H3.1-SNAP (left) and H3.3-SNAP (right) histones. Whiskers indicate the range of the intensities measured. A Mann–Whitney statistical test established the significance of the difference between siLuciferase and siJMJD1B for H3.1 (*p* < 2.6 × 10^−11^) and H3.3 (*p* < 2.2 × 10^−16^), and siLuciferase and siHIRA for H3.3 (*p* < 2.2 × 10^−16^). **d** Cell cycle profiles of HeLa cells treated with siControl or siJMJD1B synchronized with a double 2 mM Thymidine block. The graphs show DNA profiles of cells released from the Thymidine block after 0, 2, 4, 6, 8, and 10 h. The standard deviation was obtained from three independent experiments. *p < 0.05; **p < 0.01; ***p < 0.001, Student’s *t*-test. **e** Immunodetection of γH2AX from siControl and siJMJD1B treated HeLa cells after 72 h. The arrow points to micronuclei. On the right, quantitation of micronuclei observed in siControl and siJMJD1B, expressed as the mean of micronuclei per cell upon counting 100 cells of each condition. The standard deviation was obtained from 4 independent countings **p < 0.01, Student’s *t*-test

Our results show that histones H3 and H4 are arrested at early steps in the maturation cascade upon siJMJD1B. Thus, we investigated whether deposition into chromatin is also altered. We examined newly synthesized histone deposition in vivo utilizing HeLa cell lines stably expressing H3.1- and H3.3-SNAP-tagged proteins [4, 26] under siControl and siJMJD1B. As illustrated in Fig. 4a, the SNAP tag allows in vivo covalent labeling with a cell-permeable small molecule. To distinguish parental from newly synthesized histones, parental histones H3 are quenched 30 min with a non-fluorescent molecule (TMR-block). A 120-min chase time permits the synthesis of new SNAP-H3 proteins, which are subsequently labeled for 20 min with a fluorescent probe (TMR-star). As a control, we depleted the H3.3 chaperone HIRA (histone regulation A) using siRNA. Western blot analyses confirmed the reduction of HIRA and JMJD1B protein levels after siHIRA and siJMJD1B treatments, respectively (Fig. 4b). We tested the incorporation of the newly synthesized histone H3 variants H3.1 and H3.3 on chromatin. We find that H3.3 but not H3.1 chromatin deposition is decreased when HIRA levels are diminished relative to control (Fig. 4c), as previously described [26]. We then investigated the effect of siJMJD1B and found that deposition of H3.1 and H3.3 significantly decreased relative to control (Fig. 4c). We thus conclude that JMJD1B knockdown impairs histone H3 assembly onto chromatin.

### siJMJD1B cells undergo DNA damage

Given that siJMJD1B cells are affected on histone deposition, we investigated whether siJMJD1B cells have signs of cell cycle arrest and DNA damage. We first analyzed the impact of the JMJD1B knockdown on the cell cycle distribution. We synchronized either siControl or siJMJD1B HeLa cells with a double thymidine block. We then performed flow cytometry analysis of DNA content at several points up to 10 h after cell cycle release (Fig. 4d). The data revealed that upon siJMJD1B cells accumulated in S-phase quickly (2 h) and after 6 h there are more cells in G2/M phase, suggesting G2/M arrest due to DNA replication stress. We then performed immunofluorescence microscopy for γH2AX, a double-strand DNA damage marker. We found γH2AX foci in the siJMJD1B, but not in the control cells (Fig. 4e). Interestingly, we also observed the presence of micronuclei upon siJMJD1B, which are positive for γH2AX, suggesting that cells were able to evade the G2 damage checkpoint to form micronuclei, an indication of chromosome instability. Therefore, we conclude that the JMJD1B knockdown induces replication stress and DNA damage and, therefore that JMJD1B could be important for maintaining genome stability.

### JMJD1B gene mutations correlate with cancer incidence and genomic instability

**Fig. 5:**
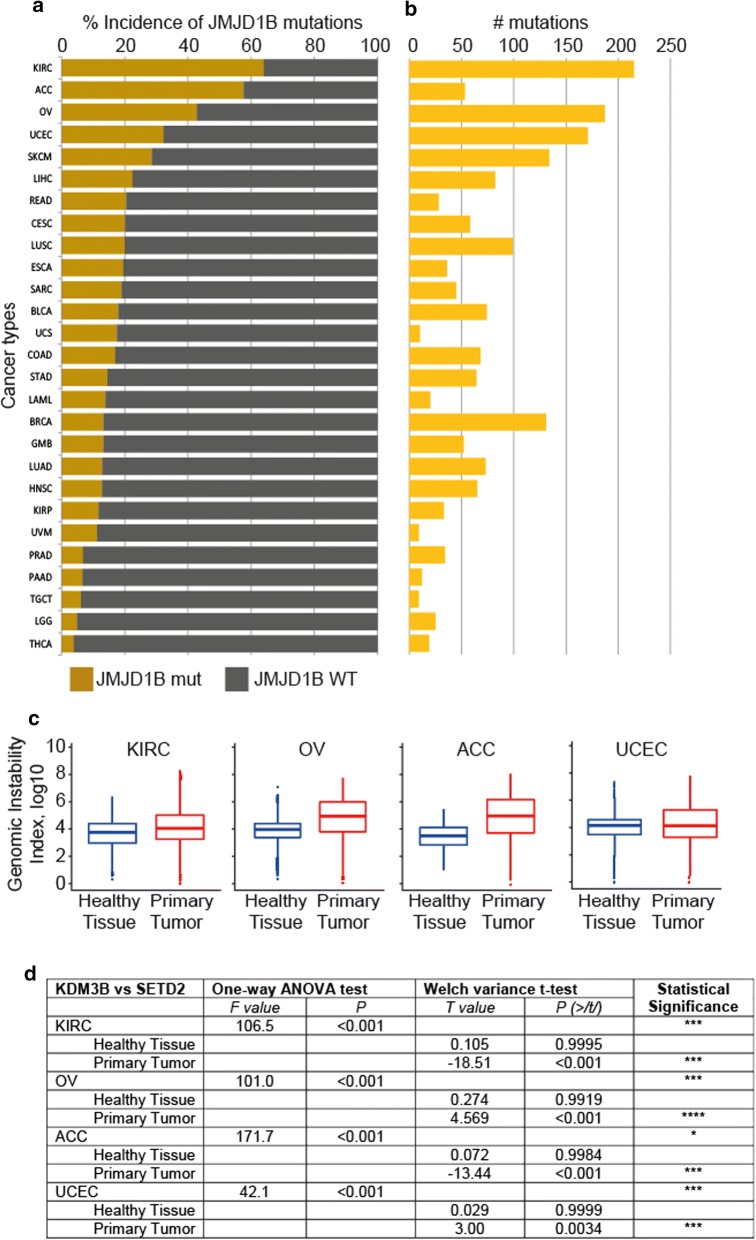
JMJD1B gene mutations correlate with high cancer incidence and with genomic instability. **a** The graph shows the incidence of JMJD1B mutations among patients with different types of cancers. Mutations include point mutations and insertion/deletions. Affymetrix data were obtained from the NIH Cancer Data Portal. **b** The graph shows the total number of mutations found per each cancer type on the JMJD1B gene. The data include point mutations and insertion/deletions. **c** Genomic instability index, expressed as log10, was calculated from the population of patients that contain mutations (point mutations and insertion/deletion) on the JMJD1B gene. The data are shown for the following cancers: KIRC, OV, ACC, and UCEC, from data derived from healthy tissues (blue) and primary tumor (red) of the same patients. Abbreviations are defined in Additional file 1: Figure S2. **d** Statistical analyses between the JMJD1B vs. SETD2 mutant populations and sample types (primary tumor vs. normal tissue) in the four cancers of interest

To investigate whether the observed defects in the maturation of newly synthesized histones H3 and H4 upon siJMJD1B could have an impact in genomic instability, we assessed the occurrence of mutations in JMJD1B gene among cancer patients in the NIH Cancer Data Portal. We first evaluated the incidence of point mutations and INDELS (insertions/deletions) in the JMJD1B gene among patients with different types of cancer. We observed that patients from several cancers have a rate of occurrence of JMJD1B mutations higher than 20% (Fig. 5a), with four cancer types (KIRC (kidney renal clear cell carcinoma*)*, ACC (adrenocortical carcinoma), OV (ovarian cancer) and UCEC (uterine corpus endometrial cancer) displaying high incidence of JMJD1B mutations (> 30%). We also scored the number of mutation events in the gene of interest per cancer (Fig. 5b). These ranged between a total of 215 in the case of KIRC patients and less than 10 in UVM (uveal melanoma) and TGCT (testicular germ cell tumor) patients. While KIRC patients displayed the highest number of different INDEL events in the JMJD1B gene, UCEC patients showed the highest diversity of point mutations (Additional file 1: Figure S2).

**Fig. 6:**
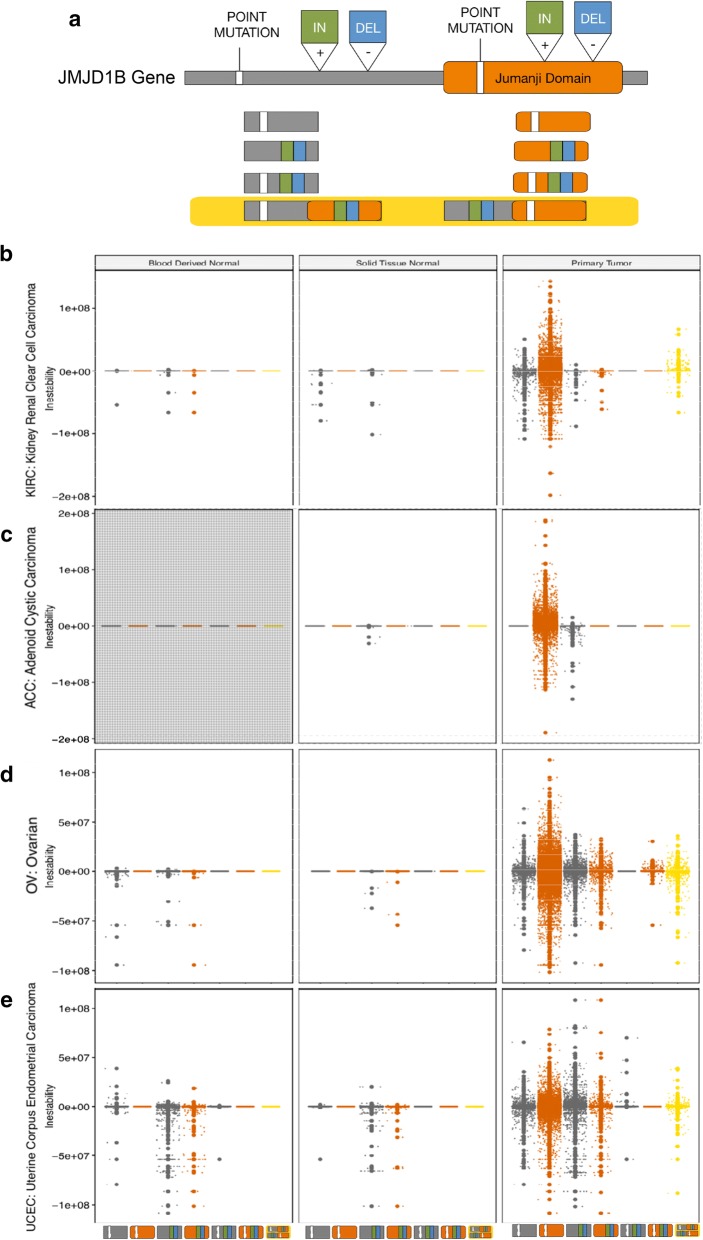
Genomic instability in patients with cancer having high incidence of mutations in the JMJD1B gene. Mutations in JMJD1B were mapped in the scheme in panel A according to their location in the gene with respect to the Jumanji domain as colored boxes: point mutations (white), insertions (IN: green) and deletions (DEL: blue). When mutations occurred in both the Jumanji domain (orange) and regions of the gene outside the domain (gray), the scheme is highlighted in yellow, regardless of the type of mutation. Color-coding used in the scheme was also applied to the symbols representing the data points in the graphs in panels B to E. The global genomic instability index was calculated as indicated in “Methods”. For each cancer type, data derived from healthy tissue (blood and/or solid tissue samples) and primary tumor biopsies was analyzed in the JMJD1B-mutants patient population. The cancer datasets considered were: KIRC (kidney renal clear cell carcinoma), ACC (adrenocortical carcinoma), OV (ovarian cancer) and UCEC (uterine corpus endometrial cancer). Missing datasets are highlighted in gray

We next investigated whether mutations in the JMJD1B gene correlated with increased genomic instability levels in cancer, using as a proxy the copy number variation available for each dataset. For this, we selected patient samples from the four types of cancers with the highest incidence of JMJD1B mutations and calculated the global genomic instability index (see “Methods”). For each cancer type we analyzed the data derived from healthy tissue (blood and/or solid tissue samples) and primary tumor biopsies in the JMJD1B mutated patient population (Fig. 5c and Additional file 1: Figure S3). In all the genomes of patients from the four cancers we observed significantly higher genomic instability in primary tumors with respect to healthy tissue samples. Interestingly, we observed increased genomic instability in the primary tumor samples for the four cancers examined, when mutations occurred in the Jumanji domain compared to other positions in the gene (Fig. 6). Previous reports showed that tumors from patients with SETD2 gene mutations have low levels of genomic instability [29], thus we retrieved a subset of JMJD1B wild type patients with mutations in SETD2 from the four cancers types alluded above and compared the genomic instability between the two populations (SETD2 mutants vs. JMJD1B mutants) using one-way ANOVA tests. The analysis of variance showed that the effect of mutations in JMJD1B on genomic instability was significant (Fig. 5d). Variance in the data, as well as the maximum instability values observed (absolute values), were higher in the JMJD1B mutant population than in the SETD2 mutants, in all four cancers analyzed. These results indicate that JMJD1B mutations positively correlate with genomic instability in the cancers analyzed, and suggest that downstream effects, such as defects in the newly synthesized histone H3 maturation, could trigger the observed genomic instability.

## Discussion

**Fig. 7:**
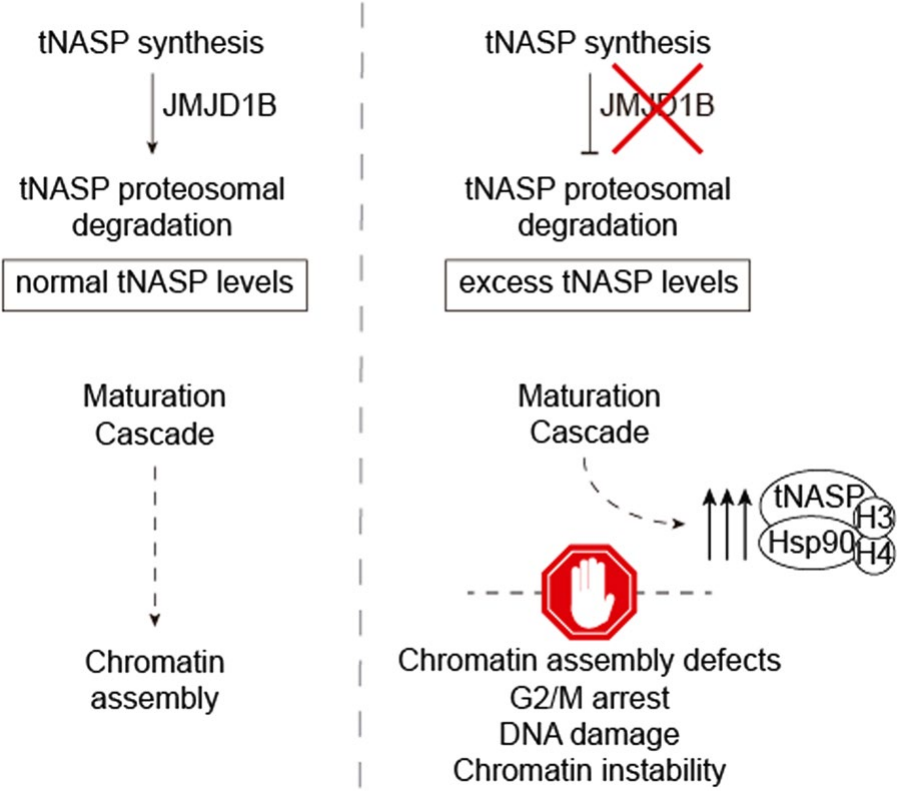
Model of the role of JMJD1B in histone H3 and H4 processing pathway. We propose that JMJD1B regulates the degradation of tNASP, thus in the absence of JMJD1B, tNASP is not degraded by the proteasome and its protein level increases. As a consequence, histones H3 and H4 accumulate early in the maturation pathway, leading to chromatin assembly defects, which could impact into G2/M arrest, DNA damage and genome instability

The assembly of eukaryotic DNA into nucleosomes represents the first level of chromosome organization, in which histone proteins contribute to chromatin structure and regulate the accessibility of factors to the underlying DNA. Proper chromatin structure and fine-tuning histone levels are connected and have crucial roles in maintaining viability and genomic stability. Here, we investigated how JMJD1B contributes to the maturation of newly synthesized histones H3 and H4 to ensure an adequate supply of histones in the cell. We propose the model illustrated in Fig. 7. Upon decreasing the levels of JMJD1B, tNASP protein levels are accumulated. This leads to the accumulation of histones H3 and H4 at early steps of the maturation cascade associated with Hsp90, Hsc70 and tNASP. As a consequence, defects in the flow of histones to chromatin assembly occur, which might contribute to G2/M arrest, and DNA damage, suggesting genomic instability. Support for this model came from the fact that mutations in the JMJD1B gene correlates with an increased genomic instability in cancer patients. Thus, our data indicate that the proper maturation of newly synthesized histones H3 and H4 is pivotal to warrant an adequate histone supply to the nucleus for chromatin assembly ensuring genome stability.

### Fine-tuning histone levels

Histone levels are regulated at several steps, and a fine-tuning is necessary since both high and low levels are dangerous for the cell. Many groups have shown that histone amounts are regulated at the level of transcription, mRNA stability, translation, and histone degradation [21]. Here, we propose another level of regulation that has been under-appreciated and that involves the maturation of newly synthesized histones. In the maturation cascade, histones H3 and H4 are processed to get their correct folding and to establish post-translation modifications. If this maturation is hindered, then histones are defective, which turns into failures in the histone supply. Thus, any way that affects the histone supply to chromatin impacts into chromatin organization. For example, the absence of the histone chaperone Asf1 in yeast increases chromatin accessibility and leads to genomic instability and sister chromatid exchange [25, 34]. As discussed previously, depletion of histones correlates with genomic instability [12, 24]. Here, we showed that by affecting the levels of the histone chaperone tNASP, newly synthesized histones are stalled at early steps of the maturation cascade.

### JMJD1B is necessary for genome stability

JMJD1B (KDM3B or JHDM2B) is a member of the JMJD1 family that also includes JMJD1A and JMJD1C, and shows histone H3K9me1/me2 [2, 12] and H4R3me2 [16] demethylase activity mainly towards gene-dense regions of chromosomes [15]. It is involved in transcriptional activation [12, 16]. Knockout mice are viable, although females and males are infertile and display cell growth defects [17, 18], as well as defects in hematopoiesis [16]. However, JMJD1A and JMJD1B double mice knockout is embryonically lethal [15]. JMJD1A/JMJD1B knockout correlates with increased levels of H3K9me2/3 in euchromatic regions of the genome, suggesting that JMJD1A and JMJD1B proteins play a role in establishing the appropriated H3K9 methylated state at early embryogenesis [15]. It is worth pointing out that, although the loss in the newly synthesized histone pools upon reducing JMJD1B levels might explain the increase DNA damage observed, we cannot rule out that additional factors could contribute to DNA damage, giving the nuclear functions of JMJD1B. Our results indicate another type of regulation achieved by JMJD1B, which operates at the level of the maturation of newly synthesized histones on the cytoplasm. Future work will focus on further elucidating the mechanism by which JMJD1B regulate tNASP protein levels.

Interestingly, previous reports suggested that JMJD1B is a tumor suppressor [10, 35]. Our analyses on cancer patients show high incidence of JMJD1B gene mutations in several types of cancers. In addition, our results also show a correlation between JMJD1B gene mutations and genomic instability. Most of the mutations in cancer patients corresponded to insertions and deletions and likely loss of function. We propose that the deregulation in the cytosolic processing of the newly synthesized histones H3/H4, that lead to defects on the histone supply to chromatin assembly, contributes to the genomic instability observed on JMJD1B mutations. Our work highlights the importance of the correct metabolism of newly synthesized histones and finds a new potential therapeutic target to restore genome integrity.

## Conclusions

We revealed the demethylase JMJD1B as a novel player in the histone maturation cascade that contributes to regulate histone supply by controlling the histone chaperone NASP protein levels. Furthermore, we find a high rate of JMJD1B mutations in cancer patients, and a correlation with genomic instability. Thus, our data point to a novel mechanism where JMJD1B is a key player to ensure a proper supply of H3 and H4 to maintain genome integrity.

## Methods

### Antibodies

Asf1 (Dr. Almouzni), β-actin (Sigma Aldrich A5316), Flag (Sigma Aldrich F3165), γH2AX (Abcam ab2893), HA (Sigma clone 12CA5), HAT1 (Abcam ab12164), HIRA (Abcam ab20655), Histone H3 (Abcam ab7834), H3ac (Merck Millipore, 06-599), Histone H4 (Abcam ab10158), H4K12ac (Merck Millipore 07-595), Hsc70 (Abcam ab19136), Hsp90 (Santa Cruz sc-7947), Importin4 (Abcam ab283887), JMJD1B (Cell Signaling Technology 2621S), NASP (Dr. Almouzni), Topo I (Santa Cruz sc-32736), SetDB1 (Abcam ab12317).

### Extract preparation

HeLa cells were cultured in Dulbecco’s modified Eagle medium (DMEM) (GE healthcare Life Sciences SH3008102) supplemented with 10% fetal bovine serum (Gibco 16000044). Cytosolic and nuclear extracts were prepared as previously described [6]. In brief, HeLa cells were ice-cold PBS washed, and trypsinized. Cell pellet was resuspended in hypotonic lysis buffer (10 mM Tris, pH 7.9, 1.5 mM MgCl_2_ 10 mM KCl, 0.5 mM DTT, 0.2 mM PMSF) and incubated for 10 min at 4 °C, after which it was centrifuged at 10,000 rpm for 10 min. Pellet was resuspended in hypotonic lysis buffer and homogenized with 10 strokes with the type B Dounce. Homogenate was centrifuged at 10,000 rpm for 10 min and the supernatant (cytosolic extract) was collected and proteins quantified using Bradford assay.

### siRNA treatment

Either 30 nM of human JMJD1B siRNA duplex (Santa Cruz, sc-91707), 10 nM of human HIRA siRNA duplex, 20 nM of human NASP siRNA duplex (Santa Cruz, sc-78745), or negative control siRNA (Ambion, Silencer Negative Control #1 siRNA, AM4611) were transfected on HeLa cells with Lipofectamine 2000 (Invitrogen) for 72 h, according to the manufacturer’s instructions.

### mRNA analysis

HeLa cells treated with either siControl or siJMJD1B for 72 h were lysed and RNA extracted using the RNeasy^®^ Mini Kit (Qiagen). cDNA was synthesized using GoScript™ Reverse Transcription System (Promega), according to the manufacturer´s instructions. qPCR was performed in a real-time PCR Cycler Rotor Gene Q (Qiagen) using KAPA SYBR^®^ FAST qPCR Master Mix (2×) (Kapa Biosystems). The following primers were used: GAPDH: forward: 5′-AGAAGGCTGGGGCTCATTTG-3′, reverse: 5′-AGGGGCCATCCACAGTCTTC-3′; HIST1H3A: forward: 5′-GTCACTATCATGCCCAAGGA-3′, reverse: R: 5′-GACCGTCAGAGAGACCACAG-3′; HIST1H3B: forward: 5′-GAGAAATCGCCCAAGACTTC-3′; reverse: 5′-ACCAAGTAGGCCTCACAAGC-3′; HIST1H3J: forward: 5′-TGCCATTTCAGCGCCTGGTG-3′, reverse: 5′-CACGGATACGACGCGCAAGC-3′; HIST2H3C: forward: 5′-GCTACCAGAAGTCCACGGAG-3′, reverse: 5′-GATGTCCTTGGGCATAATGG-3′; tNASP: forward: 5′-TGAGGAAGCAAGGGAAGAGT-3′, reverse: 5′-CCACCCTTCTCCATTTCACT-3′; t/sNASP: 5′-AAAGGAACTGCTACCCGAAA-3′, reverse: 5′-GACTGAAGAGCCTCCTCCAC-3′; JMJD1B, forward: 5′-TGCTGTTCGCGGACACTG-3′, reverse: 5′-CAAAGATCGCTAGGTCCTGGG-3′. For relative quantification, mRNA expression was normalized by comparison with GAPDH mRNA expression using the ddCT method.

### Half-life tNASP

4 × 10^6^ cells were seeded on 15-cm plates and transfected the next day with siControl or siJMJD1B. Next day, cells were collected and seeded on 6-well plates (0.25 × 10^6^ cells per well). Next day, cells were incubated with 100 μg/mL cycloheximide (CHX) for 0, 0.5, 1, 3, 5 and 7 h. Total cell extracts were immediately prepared incubating the cell pellet in RIPA buffer (20 mM Tris–HCl pH 8, 150 mM NaCl, 1% sodium deoxycholate, 0.1% SDS, 1 mM EDTA, 10 μg/mL leupeptin, 10 μg/mL aprotinin, 10 μg/mL pepstatin, 100 μM PMSF) and the protein levels over time were evaluated by Western blot.

### Inhibition of protein degradation

0.25 × 10^6^ cells were seeded on 6-well plates the day before treatment and then incubated with 100 µg/mL CHX or 100 µg/mL CHX plus 50 µM MG132 or 20 mM NH_4_Cl + 200 µM leupeptin for 7 h. DMSO was used as vehicle control. Total cell extracts were immediately prepared incubating the cell pellet in RIPA buffer and analyzed as described above.

### Size-exclusion chromatography

0.2 mg of cytosolic extracts derived from siControl or siJMJD1B treated HeLa cells were loaded onto a GE Healthcare Superdex 200 10/300 GL column equilibrated with a buffer containing 150 mM KCl plus 0.05% NP40. Fractions of 0.5 mL were collected at a flow rate of 0.5 mL/min. Protein standards (Amersham Pharmacia) were fractionated under similar conditions to estimate the complexes size.

### Immunoprecipitation assay

HeLa cells expressing Flag-H3.1 at low levels [19] were transfected with either siControl or siJMJD1B. Seventy-two hours after, we prepared cytosolic extracts and incubated 1.5 mg of siControl or siJMJD1B derived cytosolic extracts with 10 μL of anti-Flag antibodies conjugated with agarose beads, in a buffer containing 150 mM KCl and 0.05% NP40. The beads were washed three times and the bound proteins eluted with 0.2 mg of Flag-peptide. Western blot analyses were performed with the immunoprecipitated samples.

### Histone deposition assay

The assay was performed on HeLa cells as previously described [26]. In brief, 10 μM of SNAP-Cell Block (Biolabs) were added for 30 min to quench SNAP-tag activity and then performed a 2 h chase step in complete medium. After in vivo labeling with SNAP-Cell TMR-Star (Biolabs) for 20 min, cells were pre-extracted with Triton before fixation to allow the visualization of new incorporated H3-SNAP by fluorescent microscopy. Quantification of mean fluorescence intensity was done as previously described [4, 26] with minor modifications. EdU was added during pulse stage allowing visualization of replicating cells with Click-It technology (Life Technologies). For H3.1 cell line, only EdU-positive nuclei were considered for TMR fluorescence quantification whereas all the nuclei were quantified for H3.3.

### Cell cycle analysis

5.5 × 10^6^ HeLa cells were plated and transfected with siRNA, as described above. Twenty-four hours after transfection, 1.1 × 10^6^ cells were re-plated, and 2 mM thymidine was added to the cells. Sixteen hours later, the medium was changed to remove thymidine. Nine hours later 2 mM thymidine was added for another 16 h. Medium was changed to allow the release of the cells for up to 10 h. Cells were then collected and fixed with 70% ethanol overnight at − 20 °C. Then cells were incubated overnight at 4 °C with 40 µg/mL RNase A. The DNA content was analyzed by flow cytometry, staining the DNA with 40 µg/mL propidium iodide (Life Technologies). Histograms obtained were analyzed by FlowJo software (Vx 0.7, Tree Star) and the percentage of cells in G1, S or G2/M phases was analyzed in GraphPad Prism 7. Cells with DNA content less than 2 N and over 4 N were included in the analysis.

### Immunofluorescence

HeLa cells seeded on cover glasses were transfected with siRNAs as described above. Seventy-two hours later, cover glasses were washed 3 times with PBS, and fixed with 2% PFA for 20 min. Covers were then washed 3 times with PBS, and cells were permeabilized with 0.2% Triton X-100 for 10 min. After washing the cells 3 times with PBS, cells were blocked with 5% BSA, 0.1% Tween–PBS. Covers were incubated for 45 min in humid chamber with anti-γH2AX and then washed 3 times with 0.1% Tween–PBS, and incubated for 30 min with secondary antibody anti-rabbit/FITC. After 3 washes with 0.1% Tween–PBS, covers were incubated for 5 min with DAPI, washed 3 times with 0.1% Tween–PBS and 2 times with PBS. Covers were mounted over glass slides and γH2AX foci were detected by epifluorescence.

### Genomic instability index calculation

To evaluate genomic instability in cancer patients, we used Affymetrix SNP 6.0 array-based CNV (Copy Number Variation) data, accessible from the National Cancer Institute (NIH) website (https://portal.gdc.cancer.gov). All publically available CNV files (in “Copy Number Segment” format) from cancer patients with known mutations in the JMJD1B gene or SETD2 (27 cancers in total) were retrieved from the repository. In addition, the files containing cancer/patient/sample metadata and the MAFFT files containing information on all associated point mutations were downloaded and parsed into a single table for further calculations. To extract a high-confidence data from the CNV files, segment mean signal intensity thresholds of 0.3 (for copy number gain) and − 0.3 (for copy number loss) were utilized as recommended by Kim and colleagues (Kim et al. 2016), and remaining data were transformed into CNV values (https://docs.gdc.cancer.gov/Data/Bioinformatics_Pipelines/CNV_Pipeline/). This data was used to derive the genomic instability index LDR (Length of Destabilized Regions) according to the following equation:

$${\text{LDR }} = {\text{ segment}}\_{\text{length }}* \, \left( {{\text{CNV }}{-}{ 2}} \right),$$

where segment length corresponds to the length of the region destabilized in base pairs and the CNV is corrected to exclude the normal diploid dosage. Insertions events (IN) scored values of GII > 0 (positive sign), while deletions (DEL) scored GII < 0 (negative sign). Absence of INDELs resulted in a genomic instability values of GII equal to zero. Differences between the two mutant populations of interest (JMJD1B vs. SETD2) and between sample types (primary tumor vs. normal tissue) were assessed for each cancer through a one-way analysis of variance (ANOVA), the Tukey HSD (honest significant differences) a posteriori multiple pairwise-comparison between groups and the mean differences comparison for samples with uneven variance using the Welch *t*-test. Statistical analysis of the data was performed with the R Package (modules ANOVA and aov) (https://www.r-project.org/).

## Supplementary information

**Additional file 1: Figure S1.** Mass spectrometry (MS) data of enriched SetDB1 complex isolated from cytosolic HeLa cell extracts, indicating the number of peptides identified and the function for each protein. **Figure S2.** (A, C) The graph shows the incidence of either JMJD1B point (A) or INDEL (insertion/deletion) (C) mutations among patients with different types of cancers. Affymetrix data was obtained from the NIH Cancer Data Portal (B, D) The graph shows the total number of either point (B) or INDEL (D) mutations found per each cancer type on the JMJD1B gene. TCGA Project Abbreviations are as follows, ACC: Adrenocortical Carcinoma; BLCA: Bladder Urothelial Carcinoma; BRCA: Breast Invasive Carcinoma; CESC: Cervical Squamous Cell Carcinoma and Endocervical Adenocarcinoma; COAD: Colon Adenocarcinoma; ESCA: Esophageal Carcinoma; GBM: Glioblastoma Multiforme; HNSC: Head and Neck Squamous Cell Carcinoma; KIRC: Kidney Renal Clear Cell Carcinoma; KIRP: Kidney Renal Papillary Cell Carcinoma; LAML: Acute Myeloid Leukemia; LGG: Brain Lower Grade Glioma; LIHC: Liver Hepatocellular Carcinoma; LUAD: Lung Adenocarcinoma; LUSC: Lung Squamous Cell Carcinoma; OV: Ovarian Serous Cystadenocarcinoma; PAAD: Pancreatic Adenocarcinoma; PRAD: Prostate Adenocarcinoma; READ: Rectum Adenocarcinoma; SARC: Sarcoma; SKCM: Skin Cutaneous Melanoma; STAD: Stomach Adenocarcinoma; TGCT: Testicular Germ Cell Tumors; THCA: Thyroid Carcinoma; UCEC: Uterine Corpus Endometrial Carcinoma; UCS: Uterine Carcinosarcoma; UVM: Uveal Melanoma. **Figure S3.** Genomic instability index was calculated from the population of patients that contain mutations (point mutations and insertion/deletion) on the JMJD1B gene. The data is shown for the following cancers: KIRC, OV, ACC, and UCEC, from data derived from healthy tissues (blue) and primary tumor (red) of the same patients.

## Data Availability

Not applicable.

## References

[1] Alvarez F, Munoz F, Schilcher P, Imhof A, Almouzni G, Loyola A (2011). Sequential establishment of marks on soluble histones H3 And H4. J Biol Chem.

[2] Brauchle M, Yao Z, Arora R, Thigale S, Clay I, Inverardi B, Fletcher J, Taslimi P, Acker MG, Gerrits B, Voshol J, Bauer A, Schubeler D, Bouwmeester T, Ruffner H (2013). Protein complex interactor analysis and differential activity of KDM3 subfamily members towards H3K9 methylation. PLoS ONE.

[3] Campos EI, Fillingham J, Li G, Zheng H, Voigt P, Kuo WH, Seepany H, Gao Z, Day LA, Greenblatt JF, Reinberg D (2010). The program for processing newly synthesized histones H3.1 and H4. Nat Struct Mol Biol.

[4] Clement C, Vassias I, Ray-Gallet D, Almouzni G (2016). Functional characterization of histone chaperones using snap-tag-based imaging to assess de novo histone deposition. Methods Enzymol.

[5] Cook AJ, Gurard-Levin ZA, Vassias I, Almouzni G (2011). A specific function for the histone chaperone NASP to fine-tune a reservoir of soluble H3–H4 in the histone supply chain. Mol Cell.

[6] Dignam JD, Lebovitz RM, Roeder RG (1983). Accurate transcription initiation by RNA polymerase II in a soluble extract from isolated mammalian nuclei. Nucleic Acids Res.

[7] Grover P, Asa JS, Campos EI (2018). H3–H4 histone chaperone pathways. Annu Rev Genet.

[8] Gurard-Levin ZA, Quivy JP, Almouzni G (2014). Histone chaperones: assisting histone traffic and nucleosome dynamics. Annu Rev Biochem.

[9] Hammond CM, Stromme CB, Huang H, Patel DJ, Groth A (2017). Histone chaperone networks shaping chromatin function. Nat Rev Mol Cell Biol.

[10] Hu Z, Gomes I, Horrigan SK, Kravarusic J, Mar B, Arbieva Z, Chyna B, Fulton N, Edassery S, Raza A, Westbrook CA (2001). A novel nuclear protein, 5qnca (Loc51780) is a candidate for the myeloid leukemia tumor suppressor gene on chromosome 5 band Q31. Oncogene.

[11] Jasencakova Z, Scharf AN, Ask K, Corpet A, Imhof A, Almouzni G, Groth A (2010). Replication stress interferes with histone recycling and predeposition marking of new histones. Mol Cell.

[12] Kim UJ, Han M, Kayne P, Grunstein M (1988). Effects of histone H4 depletion on the cell cycle and transcription of *Saccharomyces cerevisiae*. EMBO J.

[13] Kim JY, Kim KB, Eom GH, Choe N, Kee HJ, Son HJ, Oh ST, Kim DW, Pak JH, Baek HJ, Kook H, Hahn Y, Chakravarti D, Seo SB (2012). KDM3B is the H3K9 demethylase involved in transcriptional activation of Lmo2 in leukemia. Mol Cell Biol.

[14] Kim J, Anurag M, Veeraraghavan J, Schiff R, Li K, Wang X (2016). Amplification of TLK2 Induces Genomic Instability via Impairing the G2/M Checkpoint. Mol Cancer Res..

[15] Kuroki S, Nakai Y, Maeda R, Okashita N, Akiyoshi M, Yamaguchi Y, Kitano S, Miyachi H, Nakato R, Ichiyanagi K, Shirahige K, Kimura H, Shinkai Y, Tachibana M (2018). Combined loss of JMJD1A and JMJD1B reveals critical roles for H3K9 demethylation in the maintenance of embryonic stem cells and early embryogenesis. Stem Cell Rep.

[16] Li S, Ali S, Duan X, Liu S, Du J, Liu C, Dai H, Zhou M, Zhou L, Yang L, Chu P, Li L, Bhatia R, Schones DE, Wu X, Xu H, Hua Y, Guo Z, Yang Y, Zheng L, Shen B (2018). JMJD1B demethylates H4R3me2s and H3K9me2 to facilitate gene expression for development of hematopoietic stem and progenitor cells. Cell Rep.

[17] Liu Z, Chen X, Zhou S, Liao L, Jiang R, Xu J (2015). The histone H3K9 demethylase KDM3B is required for somatic growth and female reproductive function. Int J Biol Sci.

[18] Liu Z, Oyola MG, Zhou S, Chen X, Liao L, Tien JC, Mani SK, Xu J (2015). Knockout of the histone demethylase Kdm3b decreases spermatogenesis and impairs male sexual behaviors. Int J Biol Sci.

[19] Loyola A, Bonaldi T, Roche D, Imhof A, Almouzni G (2006). Ptms on H3 variants before chromatin assembly potentiate their final epigenetic state. Mol Cell.

[20] Loyola A, Tagami H, Bonaldi T, Roche D, Quivy JP, Imhof A, Nakatani Y, Dent SY, Almouzni G (2009). The Hp1alpha-Caf1-Setdb1-containing complex provides H3k9me1 for Suv39-mediated K9me3 in pericentric heterochromatin. EMBO Rep.

[21] Marzluff WF, Koreski KP (2017). Birth and death of histone mRNAs. Trends Genet.

[22] Mendiratta S, Gatto A, Almouzni G. Histone supply: multitiered regulation ensures chromatin dynamics throughout the cell cycle. J Cell Biol. 2018. 10.1083/jcb.201807179 PMC6314538 30257851

[23] Pinheiro I, Margueron R, Shukeir N, Eisold M, Fritzsch C, Richter FM, Mittler G, Genoud C, Goyama S, Kurokawa M, Son J, Reinberg D, Lachner M, Jenuwein T (2012). Prdm3 and Prdm16 are H3k9me1 methyltransferases required for mammalian heterochromatin integrity. Cell.

[24] Prado F, Aguilera A (2005). Partial depletion of histone H4 increases homologous recombination-mediated genetic instability. Mol Cell Biol.

[25] Prado F, Cortes-Ledesma F, Aguilera A (2004). The absence of the yeast chromatin assembly factor asf1 increases genomic instability and sister chromatid exchange. EMBO Rep.

[26] Ray-Gallet D, Woolfe A, Vassias I, Pellentz C, Lacoste N, Puri A, Schultz DC, Pchelintsev NA, Adams PD, Jansen LE, Almouzni G (2011). Dynamics of histone H3 deposition in vivo reveal a nucleosome gap-filling mechanism for H3.3 to maintain chromatin integrity. Mol Cell.

[27] Rivera C, Gurard-Levin ZA, Almouzni G, Loyola A (2014). Histone lysine methylation and chromatin replication. Biochim Biophys Acta.

[28] Rivera C, Saavedra F, Alvarez F, Diaz-Celis C, Ugalde V, Li J, Forne I, Gurard-Levin ZA, Almouzni G, Imhof A, Loyola A (2015). Methylation of histone H3 lysine 9 occurs during translation. Nucleic Acids Res.

[29] Rondinelli B, Rosano D, Antonini E, Frenquelli M, Montanini L, Huang D, Segalla S, Yoshihara K, Amin SB, Lazarevic D, The BT, Verhaak RG, Futreal PA, Di Croce L, Chin L, Cittaro D, Tonon G (2015). Histone demethylase Jarid1c inactivation triggers genomic instability in sporadic renal cancer. J Clin Investig.

[30] Saavedra F, Rivera C, Rivas E, Merino P, Garrido D, Hernandez S, Forne I, Vassias I, Gurard-Levin ZA, Alfaro IE, Imhof A, Almouzni G, Loyola A (2017). Pp32 and Set/Taf-Ibeta proteins regulate the acetylation of newly synthesized histone H4. Nucleic Acids Res.

[31] Sauer PV, Gu Y, Liu WH, Mattiroli F, Panne D, Luger K, Churchill MEA (2018). Mechanistic insights into histone deposition and nucleosome assembly by the chromatin assembly factor-1. Nucleic Acids Res..

[32] Singh RK, Liang D, Gajjalaiahvari UR, Kabbaj MH, Paik J, Gunjan A (2010). Excess histone levels mediate cytotoxicity via multiple mechanisms. Cell Cycle.

[33] Towbin BD, Gonzalez-Aguilera C, Sack R, Gaidatzis D, Kalck V, Meister P, Askjaer P, Gasser SM (2012). Step-wise methylation of histone H3k9 positions heterochromatin at the nuclear periphery. Cell.

[34] Tyler JK, Adams CR, Chen SR, Kobayashi R, Kamakaka RT, Kadonaga JT (1999). The Rcaf complex mediates chromatin assembly during dna replication and repair. Nature.

[35] Xu X, Nagel S, Quentmeier H, Wang Z, Pommerenke C, Dirks WG, Macleod RAF, Drexler HG, Hu Z (2018). Kdm3b shows tumor-suppressive activity and transcriptionally regulates Hoxa1 through retinoic acid response elements in acute myeloid leukemia. Leuk Lymphoma.

