# Supplementary material for: JMJD1B, a novel player in histone H3 and H4 processing to ensure genome stability
Source: Epigenetics Chromatin. 2020 Feb 18;13:6. doi: 10.1186/s13072-020-00331-1 (PMC7027290; doi:10.1186/s13072-020-00331-1)
Supplement: Supplementary file 1 — Additional file 1: Figure S1. Mass spectrometry (MS) data of enriched SetDB1 complex isolated from cytosolic HeLa cell extracts, indicating the number of peptides identified and the function for each protein. Figure S2. (A, C) The graph shows the incidence of either JMJD1B point (A) or INDEL (insertion/deletion) (C) mutations among patients with different types of cancers. Affymetrix data was obtained from the NIH Cancer Data Portal (B, D) The graph shows the total number of either point (B) or INDEL (D) mutations found per each cancer type on the JMJD1B gene. TCGA Project Abbreviations are as follows, ACC: Adrenocortical Carcinoma; BLCA: Bladder Urothelial Carcinoma; BRCA: Breast Invasive Carcinoma; CESC: Cervical Squamous Cell Carcinoma and Endocervical Adenocarcinoma; COAD: Colon Adenocarcinoma; ESCA: Esophageal Carcinoma; GBM: Glioblastoma Multiforme; HNSC: Head and Neck Squamous Cell Carcinoma; KIRC: Kidney Renal Clear Cell Carcinoma; KIRP: Kidney Renal Papillary Cell Carcinoma; LAML: Acute Myeloid Leukemia; LGG: Brain Lower Grade Glioma; LIHC: Liver Hepatocellular Carcinoma; LUAD: Lung Adenocarcinoma; LUSC: Lung Squamous Cell Carcinoma; OV: Ovarian Serous Cystadenocarcinoma; PAAD: Pancreatic Adenocarcinoma; PRAD: Prostate Adenocarcinoma; READ: Rectum Adenocarcinoma; SARC: Sarcoma; SKCM: Skin Cutaneous Melanoma; STAD: Stomach Adenocarcinoma; TGCT: Testicular Germ Cell Tumors; THCA: Thyroid Carcinoma; UCEC: Uterine Corpus Endometrial Carcinoma; UCS: Uterine Carcinosarcoma; UVM: Uveal Melanoma. Figure S3. Genomic instability index was calculated from the population of patients that contain mutations (point mutations and insertion/deletion) on the JMJD1B gene. The data is shown for the following cancers: KIRC, OV, ACC, and UCEC, from data derived from healthy tissues (blue) and primary tumor (red) of the same patients. [file 13072_2020_331_MOESM1_ESM.docx]

**ADDITIONAL INFORMATION**


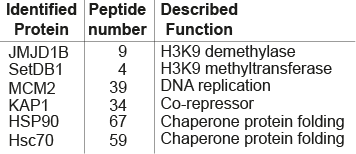


**Figure S1.** Mass spectrometry (MS) data of enriched SetDB1 complex isolated from cytosolic HeLa cell extracts, indicating the number of peptides identified and the function for each protein.


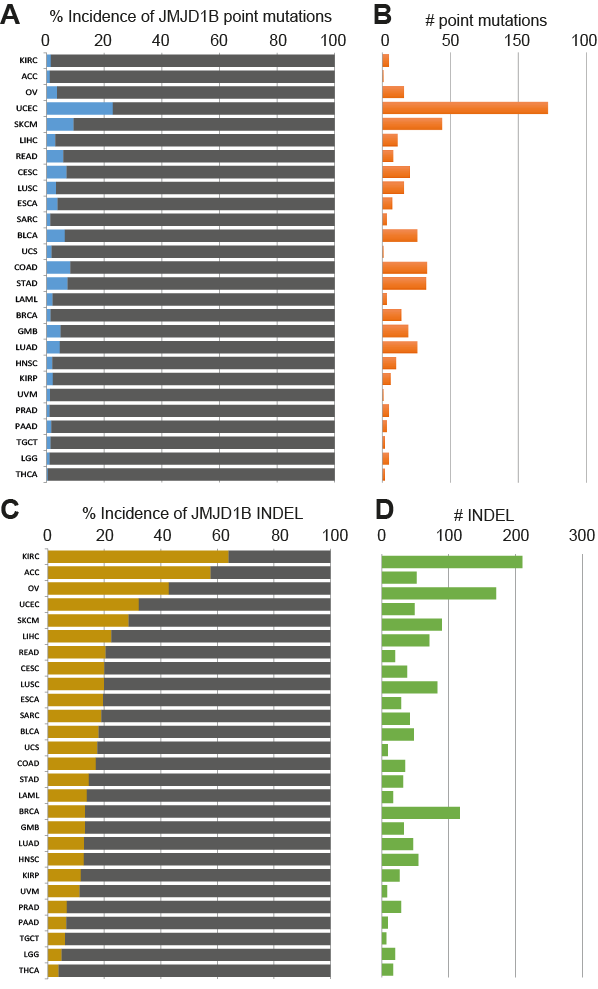


**Figure S2.** (A, C) The graph shows the incidence of either JMJD1B point (A) or INDEL (insertion/deletion) (C) mutations among patients with different types of cancers. Affimetrix data was obtained from the NIH Cancer Data Portal (B, D) The graph shows the total number of either point (B) or INDEL (D) mutations found per each cancer type on the JMJD1B gene. TCGA Project Abbreviations are as follows, ACC: Adrenocortical Carcinoma; BLCA: Bladder Urothelial Carcinoma; BRCA: Breast Invasive Carcinoma; CESC: Cervical Squamous Cell Carcinoma and Endocervical Adenocarcinoma; COAD: Colon Adenocarcinoma; ESCA: Esophageal Carcinoma; GBM: Glioblastoma Multiforme; HNSC: Head and Neck Squamous Cell Carcinoma; KIRC: Kidney Renal Clear Cell Carcinoma; KIRP: Kidney Renal Papillary Cell Carcinoma; LAML: Acute Myeloid Leukemia; LGG: Brain Lower Grade Glioma; LIHC: Liver Hepatocellular Carcinoma; LUAD: Lung Adenocarcinoma; LUSC: Lung Squamous Cell Carcinoma; OV: Ovarian Serous Cystadenocarcinoma; PAAD: Pancreatic Adenocarcinoma; PRAD: Prostate Adenocarcinoma; READ: Rectum Adenocarcinoma; SARC: Sarcoma; SKCM: Skin Cutaneous Melanoma; STAD: Stomach Adenocarcinoma; TGCT: Testicular Germ Cell Tumors; THCA: Thyroid Carcinoma; UCEC: Uterine Corpus Endometrial Carcinoma; UCS: Uterine Carcinosarcoma; UVM: Uveal Melanoma.


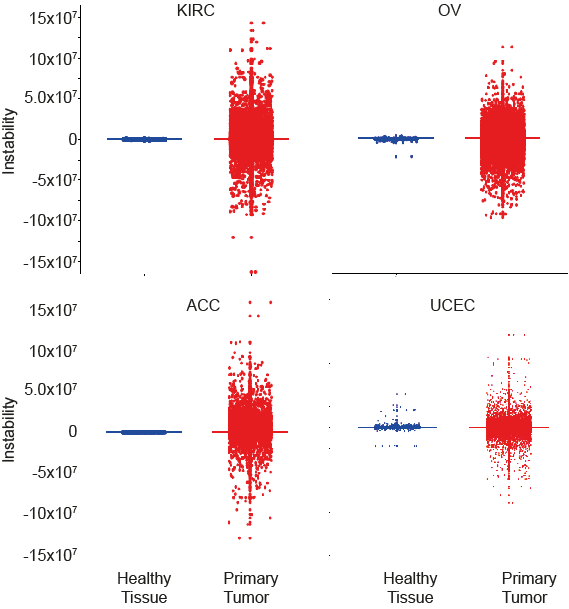


**Figure S3.** Genomic instability index was calculated from the population of patients that contain mutations (point mutations and insertion/deletion) on the JMJD1B gene. The data is shown for the following cancers: KIRC, OV, ACC, and UCEC, from data derived from healthy tissues (blue) and primary tumor (red) of the same patients.
